# Species-specific influence of powdery mildew mycelium on the efficiency of PM accumulation by urban greenery

**DOI:** 10.1007/s11356-023-28371-6

**Published:** 2023-06-23

**Authors:** Arkadiusz Przybysz, Adam Nawrocki, Ewa Mirzwa-Mróz, Elżbieta Paduch-Cichal, Kinga Kimic, Robert Popek

**Affiliations:** 1 Section of Basic Research in Horticulture, Department of Plant Protection, Institute of Horticultural Sciences, Warsaw University of Life Sciences – SGGW (WULS-SGGW), Nowoursynowska 159, 02-776 Warsaw, Poland; 2 Section of Plant Pathology, Department of Plant Protection, Institute of Horticultural Sciences, Warsaw University of Life Sciences – SGGW (WULS-SGGW), Nowoursynowska Street 159, 02-776 Warsaw, Poland; 3 Department of Landscape Architecture, Institute of Environmental Engineering, Warsaw University of Life Sciences – SGGW (WULS-SGGW), Nowoursynowska Street 159, 02-776 Warsaw, Poland

**Keywords:** Air biofiltration, Mycelium, Powdery mildew, PM accumulation, Urban greenery

## Abstract

Particulate matter (PM) is one of the most important air pollutants, especially in urban areas. The efficiency of PM biofiltration by plants depends on the morphological features of the foliage. More PM is deposited on complex leaves, covered with thick wax layer, trichomes, epidermal glands, and convex venation. Very few literature reports suggest that also the presence of mycelium of nonparasitic and saprophytic fungi positively affects the accumulation of PM on the leaves. In this work, to our best knowledge, for the first time the effect of the mycelium of the parasitic powdery mildew on the efficiency of PM accumulation by urban greenery was studied. Uninfested and fungus-infested leaves of *Acer negundo* L., *Malus domestica* Borkh *Quercus robur* L., and *Berberis vulgaris* L. were harvested in July in the center of Warsaw city. The effect of powdery mildew infection on PM accumulation was species-specific. A higher amount of PM on leaves not infected with powdery mildew was found in *M. domestica* and *Q. robur*, while in *A. negundo* and *B. vulgaris* more PM was accumulated on leaves infected with fungus. All species (except *A. negundo*) accumulated more of the PM of 0.2–2.5-μm and 2.5–10-μm size fractions on leaves not infected with powdery mildew. One of the greatest consequences of the presence of powdery mildew mycelium on the foliage is most probably reduction of the direct involvement of waxes in PM accumulation and retention processes.

## Introduction

Air pollution is one of the greatest threats to the health and comfort of urban residents (WHO global air quality guidelines: particulate matter (PM2.5 and PM10), ozone, nitrogen dioxide, sulfur dioxide and carbon monoxide, 2021). The most dangerous air pollutant is particulate matter (PM). The negative impact on living organisms depends on physico-chemical properties of PM (Ramli et al. 2020). The smallest (under 10 μm) PM is the most dangerous to human health and wellbeing (Li and Managi 2022). Exposure to PM leads to serious problems with the inhalatory and cardiovascular systems (Yang et al. 2021; Khosravipour et al. 2022). Main sources of PM in urban areas are road traffic and individual heating (Coelho et al. 2022).

Particulate matter can be effectively removed from the atmosphere by plants through air biofiltration processes (Chen et al. 2021; Chávez-García and González-Méndez 2021; Han et al. 2022). Trees, shrubs, and herbaceous plants (meadows) have already shown great potential for air purification (Kończak et al. 2021; Przybysz et al. 2021; Vigevani et al. 2022; Popek et al. 2022). The effectiveness of air biofiltration depends mainly on morphological properties of the leaves, e.g., size, shape, structure, and amount of waxes (Wróblewska and Jeong 2021). Leaves with a complex structure (covered with a thick wax layer, epidermal glands, convex venation, and dense trichomes) accumulated the greater amount of PM than leaves with smooth surface (Weerakkody et al. 2018a, 2018b; Chávez-García and González-Méndez 2021; Chen et al. 2021). PM is also absorbed through/inside stomata (Chen et al. 2022). It was also demonstrated that the phyllosphere of leaves actively participates in the decomposition of accumulated pollutants and can have significant influence on accumulation potential of the leaves (Weyens et al. 2015; Wróblewska and Jeong 2021).

Until now, the vast majority of scientists perceived leaf-inhabiting fungi only as a biological source of PM (spores and fragments of mycelium), while not as a factor influencing the efficiency of air pollution accumulation on foliage (Grantz et al. 2003; Cai et al. 2017; Zhai et al. 2018; Ramli et al. 2020). Few literature reports suggest a possible positive effect of the presence of fungal hyphae on the leaf surface on the efficiency of PM accumulation and its further retention (iMori et al. 2015; Sánchez-López et al. 2015; Muhammad et al. 2019). Jouraeva et al. (2006) demonstrated that nonparasitic and saprotrophic sooty mold fungi play an important role in accumulation of atmospheric air pollutants, including PM. Leaves of *Tilia* × *euchlora* K. Koch infected by sooty mold fungi trapped significantly higher amounts of polycyclic aromatic hydrocarbons (PAHs) and heavy metals (HM) than leaves of the same species but not affected by molds and can play an essential role in the removal of many toxins associated with atmospheric PM. Moreover, most probably sooty mold fungi can participate in ezymatic degradation of PM-bound PAHs. The authors also suggest that in the absence of sooty mold fungi, physico-chemical properties of waxes, rather than their amounts, play an important role in accumulation of PM on leaves (Jouraeva et al. 2006). Mori et al. (2015) demonstrated that presence of algae and/or fungi on *Picea sitchensis* (Bong.) Carrière and *Pinus sylvestris* L. needles may increase the amount of the accumulated PM on foliage. Observations carried out on environmental scan electronic microscope showed an unidentified fungal mycelium and PM bound to it on the needle surfaces of both species (Mori et al. 2015). Increased PM retention on leaves covered with fungal hyphae was found by Muhammad et al. (2019). The high PM accumulation and retention capacity possibly attributed to the presence of an abundant unidentified fungal mycelium forming a wide web bounding PM was recorded in *Flaveria trinervia (Spreng.)* C. Mohr, *Dichondra argentea* Willd., *Aster gymnocephalus* (DC.) A. Gray, *Crotalaria pumila* Ortega, *Viguiera dentata* (Cav.) Spreng., Gnaphalium sp. L., and *Cuphea lanceolata* W.T. Aiton (Sánchez-López et al. 2015). The mycelium of phyllospheric fungi also participated in the increased retention of potentially different potentially toxic elements (PTEs) (Sánchez-López et al. 2015). Smith and Staskawicz (1977) observed that fungal mycelium, which becomes particularly abundant on leaf surfaces as the growing season progresses, is in intimate association with PM accumulation by plants. Finally, the ability of fungal mycelium (white oyster mushroom—*Pleurotus ostreatus* (Jacq.) P. Kumm.) to accumulate and retain PM has also been successfully used in mycelium-composite panels for atmospheric PM adsorption (Lee and Choi 2021). However, in the available literature, there is no data on the impact of plant infection by pathogenic fungi on the accumulation of PM. It is well known that among the various species of pathogenic fungi, there are species that abundantly produce mycelium on the surface of infected leaves, which include, among others, powdery mildew (Braun and Cook 2012).

Powdery mildews are a large group of common plant pathogens of major economic importance (Braun 2011; Braun and Cook 2012; Marçais and Desprez-Lousta 2014; Kimic et al. 2022). These fungi belong to the order Erysiphales (H.Gwynne-Vaughan), type Ascomycota (Caval.-Sm.). On infected plants, they develop white powdery spots or patches composed of mycelium, conidia and conidiophores. The most infected are the lower leaves but the symptoms can appear on any aboveground part of the plant. Mycelium grows mainly on the surface of plant tissues but does not penetrate them. In severe forms, individual plant organs can be completely coated with mycelium. Fungi obtain nutrients from the plants using feeding organs, called haustoria, that form in the epidermal cells (Braun 2011; Braun and Cook 2012; Marçais and Desprez-Lousta 2014; Kimic et al. 2022). As a result of the plant infection with powdery mildew, changes in the physiological functions of the diseased plants are observed (increased respiratory intensity, reduced photosynthetic intensity, disruption of the flow of water and the inorganic and organic compounds dissolved therein to transpiring organs, and disruption of the flow of assimilates) (Hajji et al. 2009; Marçais and Desprez-Lousta 2014).

Powdery mildew develops in two stages: anamorph, the stage at which the fungus reproduces asexually, and teleomorph, the stage at which the fungus reproduces sexually (Braun and Cook 2012). In the anamorph, the mycelium produces conidiophores with conidia. Depending on the species, there are 2 main types of conidiophores: Pseudoidium (conidia formed singly) and catenescent (conidia formed in genuine chains) types. In the teleomorph, the fungus forms asci with ascospores in fruiting bodies, called chasmothecia. A very important epidemiological feature of powdery mildew is the ability to infect plants over a broad range of temperatures and humidities. Conidia can germinate without water and the optimal air humidity for infection is 90–99%. The incubation period is short (about a week). Consequently, under favorable conditions, plant infections can occur on a nearly daily basis (Braun and Cook 2012). In Poland, even in summer at high temperatures, a night-time temperature drop and humidity increase are sufficient for the powdery mildew spores to germinate and infect plants, resulting in a rapid spread of the disease (Kimic et al. 2021, 2022). Shrubs and trees with symptoms of powdery mildew diseases caused by many different fungi species can be observed in parks, squares, and gardens (Kimic et al. 2021, 2022).

As outlined above, fungal mycelium most probably increases PM accumulation on plants foliage. However, knowledge on this subject is still incomplete (in most works the fungus is not even identified) and requires confirmation, especially for pathogenic fungi. Very little is known about whether fungal mycelium increases PM accumulation on foliage of every plant species or whether this effect is species-specific and depends on the leaf morphology. Therefore, in this study, we studied PM accumulation by *Malus domestica* Borkh., *Quercus robur* L., *Acer negundo* L., and *Berberis vulgaris* L. uninfected and infected by powdery mildew. In order to correctly interpret the potential impact of powdery mildew on the effectiveness of plants in air biofiltration in urban areas, the experiment was carried out in realistic conditions of a big city.

## Materials and methods

### Plant material

**Fig. 1:**
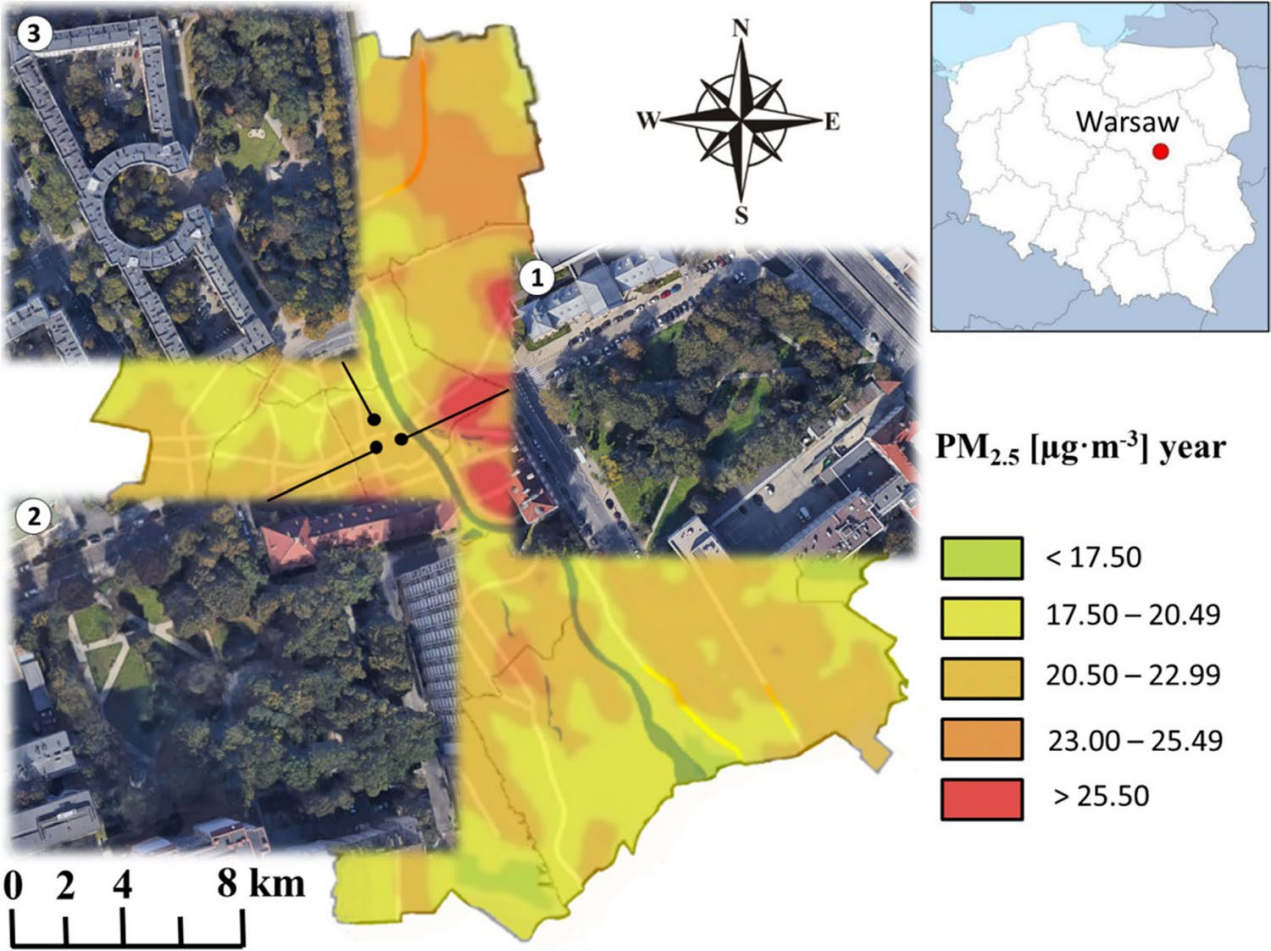
Average annual air concentration of PM_2.5_ in Warsaw (Poland). The black dots mark the locations of the study area: (1) the Square of the Matysiak Radio Family, (2) the Siberian Square, and (3) the Square of Political Prisoners of Stalinism (Przybysz et al. 2021, modified)

Plant material was harvested from trees and shrubs growing in three green squares located in the central districts of Warsaw, capital of Poland (1.86 million residents): the Square of the Matysiak Radio Family (Skwer Radiowej Rodziny Matysiaków, area: 0.82 ha), the Siberian Square (Skwer Sybiraków, area: 0.74 ha), and the Square of Political Prisoners of Stalinism (Skwer Więźniów Politycznych Stalinizmu, area: 1.34 ha) (Fig. 1). Plants growing in all three squares were exposed to high air pollution resulting primarily from their location in highly urbanized areas and next to roads with heavy traffic. The average annual concentrations of PM_10_ and PM_2.5_ in studied locations were: 33.6 μg⋅m^−3^ PM_10_ and 24.1 μg⋅m^−3^ PM_2.5_ in the Square of the Matysiak Radio Family, 30.1 μg⋅m^−3^ PM_10_ and 17.9 μg⋅m^−3^ PM_2.5_ in the Square of Political Prisoners of Stalinism, and 35.2 μg⋅m^−3^ PM_10_ and 20.3 μg⋅m^−3^ PM_2.5_ in the Siberian Square (GIOŚ 2022). These concentrations are above the annual average limits recommended by the World Health Organization established at 15 μg⋅m^−3^ for PM_10_ and 5 μg⋅m^−3^ for PM_2.5_ (WHO global air quality guidelines: particulate matter (PM2.5 and PM10), ozone, nitrogen dioxide, sulfur dioxide and carbon monoxide., 2021).

Based on the morphology, using mycological keys, literature (Braun 2011; Braun and Cook 2012; Marcinkowska 2012; Sałata 1985), Index Fungorum (Index Fungorum 2022), and the Mycobank (Mycobank Database 2023), the powdery mildew pathogens have been identified on the leaves of three tree species: *Acer negundo* L. (the Square of the Matysiak Radio Family), *Malus domestica* Borkh*.* (the Square of the Matysiak Radio Family—location 1, the Siberian Square—location 2), *Quercus robur* L. (the Square of Political Prisoners of Stalinism—location 3), and shrub: *Berberis vulgaris* L. (the Siberian Square). Leaf samples were collected in early July 2021 from mature plants in full vegetation. From each species, fully developed leaves with clearly visible etiological signs indicating their infection by a specific pathogen species (experimental group) and healthy leaves (control group) were harvested from at least four biological replicates (the biological replicate was a single plant growing in a given location). Leaves of all examined species infected by powdery mildew mostly had 40–50% of the leaf surface covered with a mycelium (bloom) of fungus; however, in order to reflect the realistic situation on the plants analytical samples contained also few leaves with a slightly lower (25–40%) and higher (50–60%) levels of infection.

The conidial spores of individual powdery mildew species were identified within the bloom. On A*. negundo* leaves *Sawadaea bicornis* (Wallr.: Fr.) Homma (type of conidia: catenescent, macro-conidia and micro-conidia), on *B. vulgaris* leaves *Erysiphe berberidis* DC. var *berberidis* (type of conidia: pseudoidium), on *M. domestica* leaves *Podosphaera leucotricha* (Ellis et Everh.) E. S. Salmo (type of conidia: catenescent), and on *Q. rubra* leaves *Erysiphe alphitoide*s (Griff. et Maubl.) U.Braun et S. Takam (type of conidia: pseudoidium) species of powdery mildew were recorded. From trees, the leaves were collected from various branches of the outer, lower (1.5–2 m, the level of human breathing) part of the crown. In the case of *B. vulgaris* shrubs, the plant material consisted of outer leaves harvested from shoots located in different parts of the plant. The leaf samples had an area of about 300 cm^2^, to avoid clogging the pores of filters during PM analysis.

### Quantitative assessment of PM and fruit wax content

The content of water-insoluble PM was examined in accordance with Dzierżanowski et al. (2011). Two categories of PM were determined: (i) water-washable from leaf surfaces (_S_PM) and (ii) retained in wax (_W_PM). The plant material was first washed for 60 s with a 200-mL distilled water, and thereafter for 30 s with 150 mL chloroform. The amount and duration of chloroform washing were determined before the experiment in order to collect accumulated PM most effectively without dissolving the top layers of the epidermis. The fractional division for both categories (_S_PM and _W_PM) was performed sequentially. The washing solutions were first sieved through a metal sieve (retention 100 μm, Haver & Boecker, Germany) and then filtered through a 10-μm paper filter (Whatman, UK, Type 91), followed by a 2.5-μm paper filter (Whatman, UK, Type 42), and finally a 0.2-μm PTFE membrane filter (Whatman, UK). The filtration was performed using a filtration set equipped with a 47-mm glass filter funnel (PALL Corp., USA) connected to a vacuum pump. Three fractions of PM were collected: (i) 10–100 μm (large), (ii) 2.5–10 μm (coarse), and (iii) 0.2–2.5 μm (fine). The sum of all PM fractions was designated as total PM. The filters were dried for 30 min at 60 °C, stabilized in the weighing room for 30 min, and weighed before and after filtration (balance XS105DU, Mettler-Toledo International Inc. and deionizer gate, HAUG, both Switzerland). The amount of wax dissolved in chloroform was assayed for each plant sample in pre-weighed beakers after chloroform evaporation. The total leaf area of each plant sample was measured on peeled skins (Image Analysis System, Skye Instruments Ltd., UK and Skye-Leaf software). The results were expressed as µg PM per cm^2^ leaf.

### Statistics

The effect of powdery mildew infestation on PM accumulation (total PM, three size fractions, two PM categories) and wax content was analyzed with Statgraphics Plus 4.1 (Statpoint Technologies Inc., Warrenton, VA, USA). The results were subjected to an analysis of variance at a significance level of *P* ≤ 0.05. The Tukey’s honest significant difference (HSD) test was used to determine the significance of differences between the means. The data are given as means with standard errors of the mean (± SE).

## Results

**Fig. 2:**
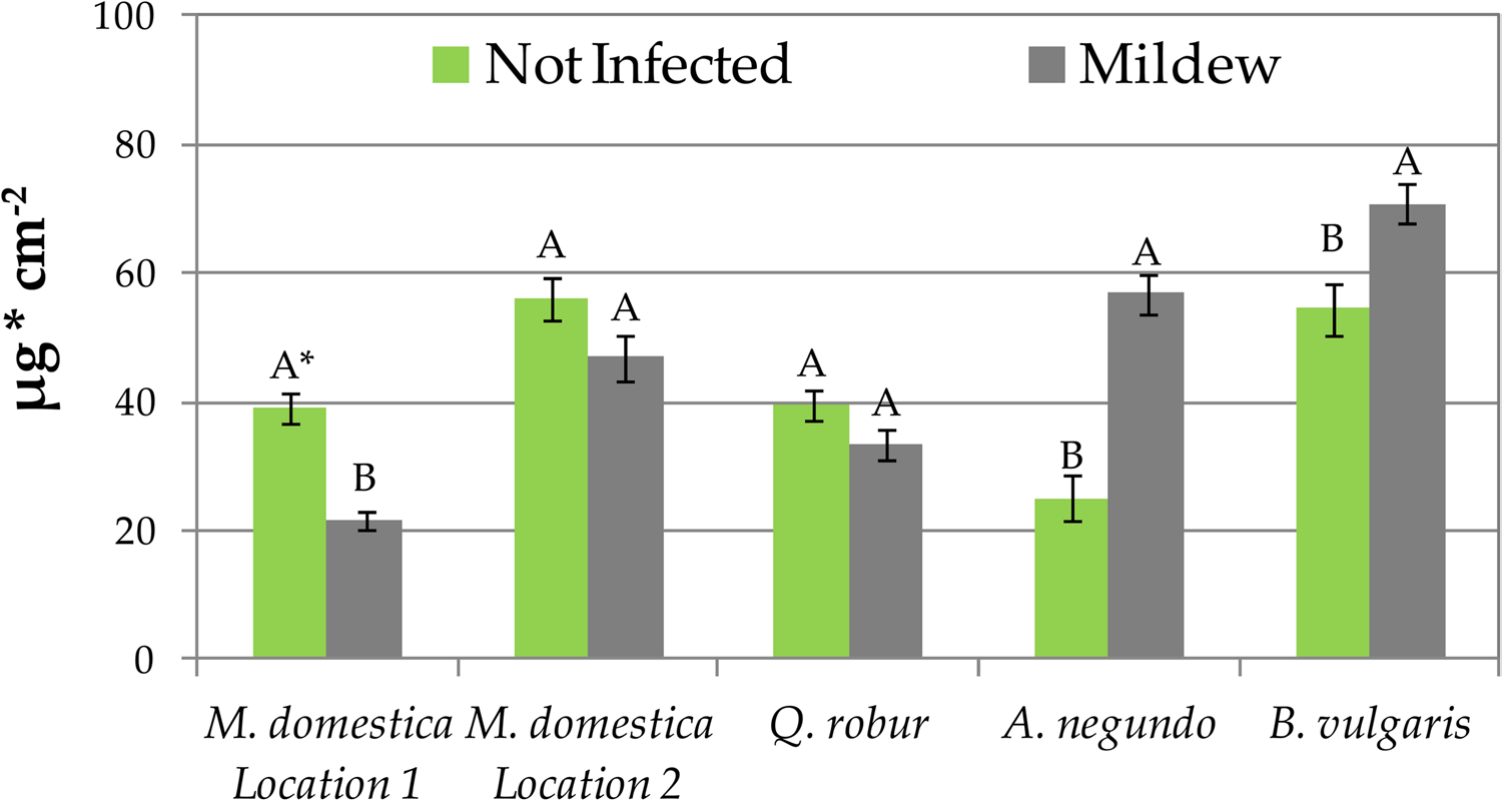
Accumulation of total PM on leaves not infected and infected with powdery mildew. Results are mean ± SE, *n* = 4 (*B. vulgaris*), 8 (*M. domestica* location 2, *Q. robur*, *A. negundo*), or 16 (*M. domestica* location 1). A single asterisk (^*^) indicates different uppercase letters indicate significant difference between leaves infected and not infected with powdery mildew at *P* < 0.05 by Tukey’s test

The impact of powdery mildew mycelium on efficiency of PM accumulation was plant species-dependent (Fig. 2). A higher amount of PM on leaves not infected with powdery mildew was found in *M. domestica* (both locations, significantly only in location 1) and *Q. robur* (insignificantly) trees, while in *A. negundo* and *B. vulgaris* significantly more PM was accumulated on leaves infected with powdery mildew. PM disposition on uninfected *M. domestica* and *Q. robur* leaves was 18–80% higher than on leaves infected with powdery mildew. The positive effect of powdery mildew on PM accumulation in *A. negundo* and *B. vulgaris* was 125% and 30%, respectively (Fig. 2).

**Fig. 3:**
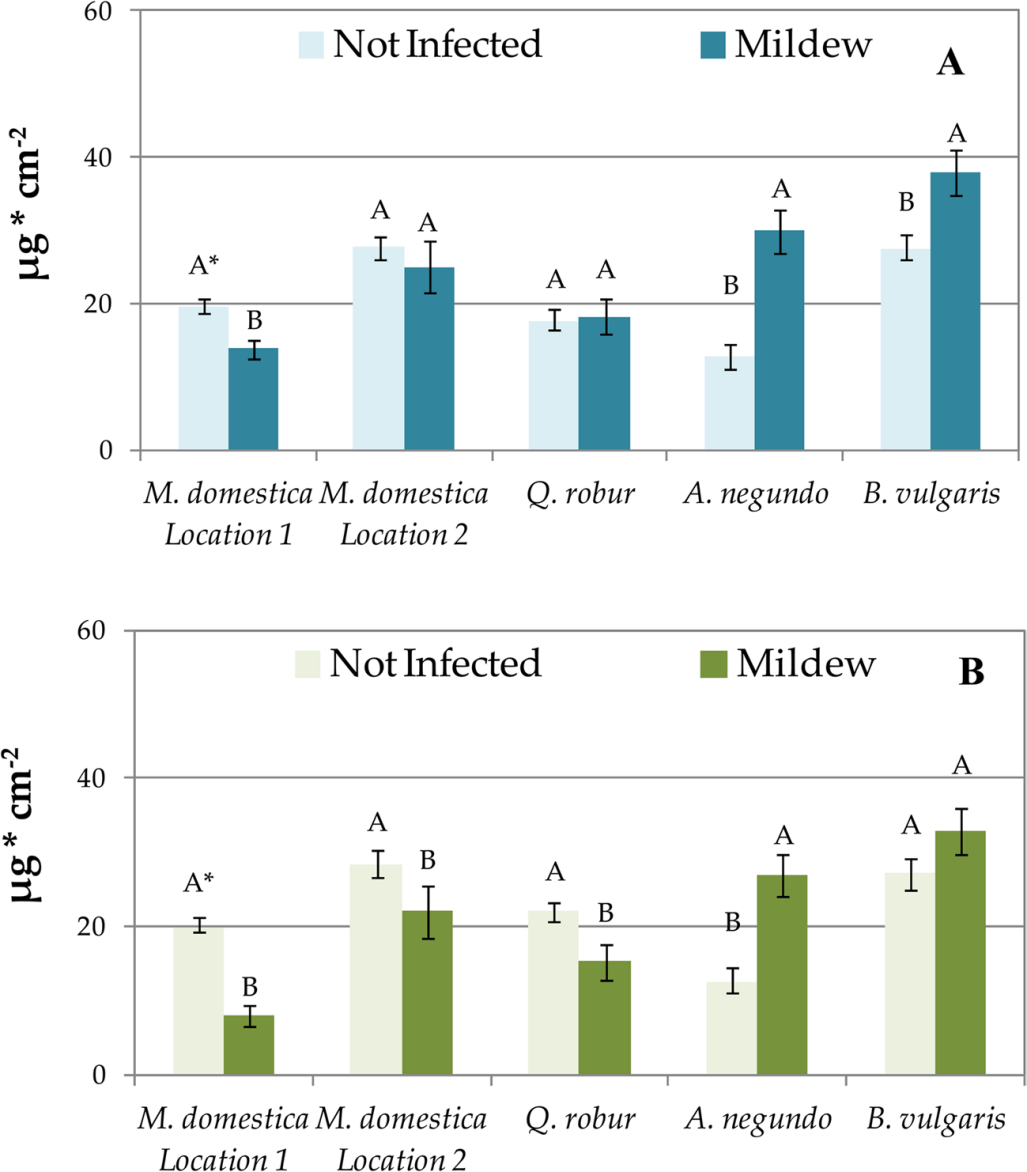
Accumulation of surface PM (sPM, **A**) and in-wax PM (wPM, **B**) on leaves not infected and infected with powdery mildew. Results are mean ± SE, *n* = 4 (*B. vulgaris*), 8 (*M. domestica* location 2, *Q. robur*, *A. negundo*), or 16 (*M. domestica* location 1). A single asterisk (^*^) indicates different uppercase letters indicate significant difference between leaves infected and not infected with powdery mildew at *P* < 0.05 by Tukey’s test

*M. domestica* leaves not infected with powdery mildew accumulated more sPM and wPM than *M. domestica* leaves infected by the fungus (Fig. 3). In the case of A. *negundo* and *B. vulgaris*, contrary results were recorded as the deposition of sPM and wPM was always higher on leaves infected with powdery mildew. Uninfected and mildew-infected *Q. robur* leaves accumulated very similar amounts of sPM, while wPM was deposited in greater amounts on non-infected leaves (Fig. 3).

**Fig. 4:**
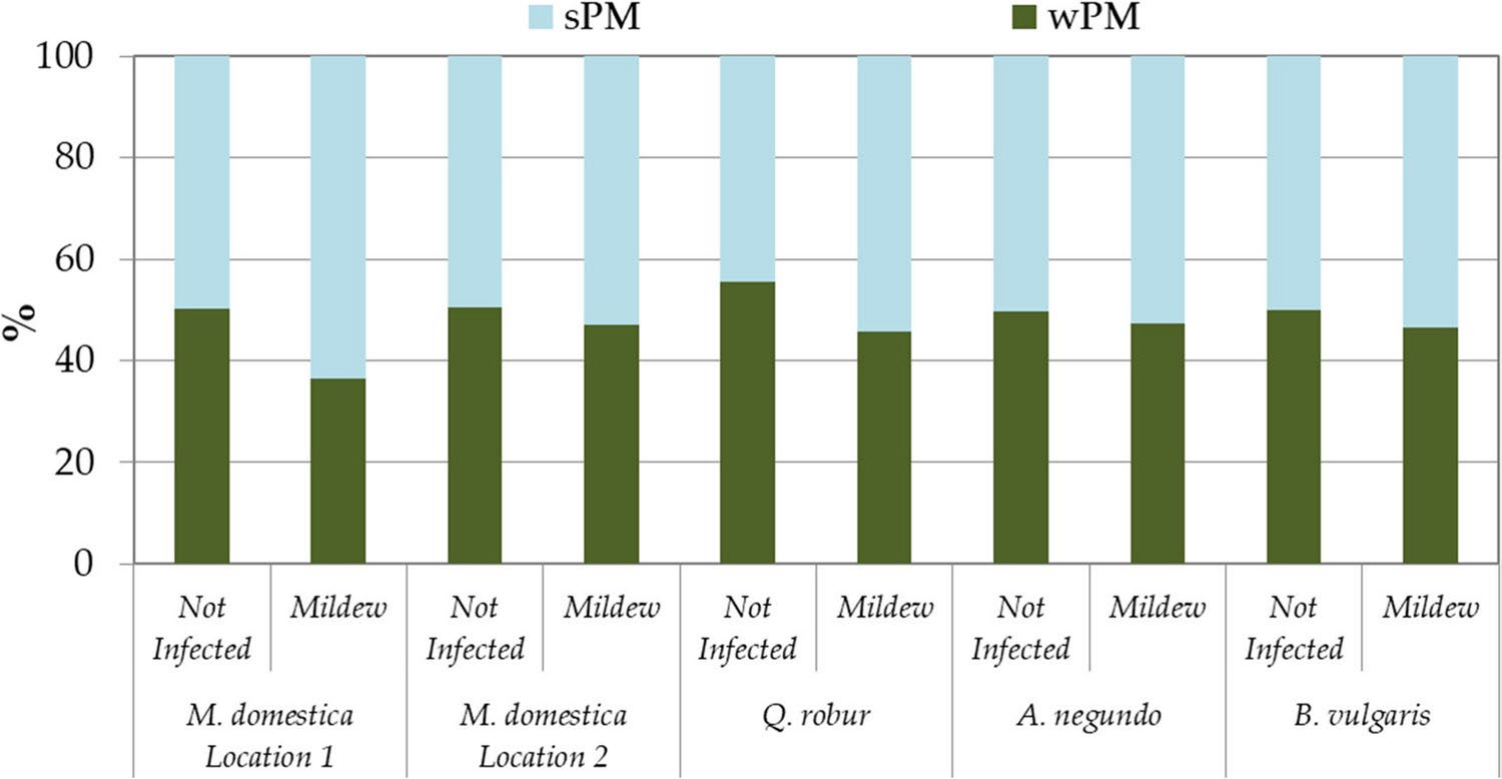
The percentage share of surface PM (sPM) and in-wax PM (wPM) on leaves not infected and infected with powdery mildew. Results are mean, *n* = 4 (*B. vulgaris*), 8 (*M. domestica* location 2, *Q. robur*, *A. negundo*), or 16 (*M. domestica* location 1)

In *M. domestica* and *Q. robur*, leaves uninfected and infected with powdery mildew differed in the share of accumulated sPM and wPM (Fig. 4). The mildew-infected leaves accumulated more PM as sPM than not infected leaves. In the case of *A. negundo* and *B. vulgaris*, the shares of sPM and wPM were very similar (Fig. 4).

**Fig. 5:**
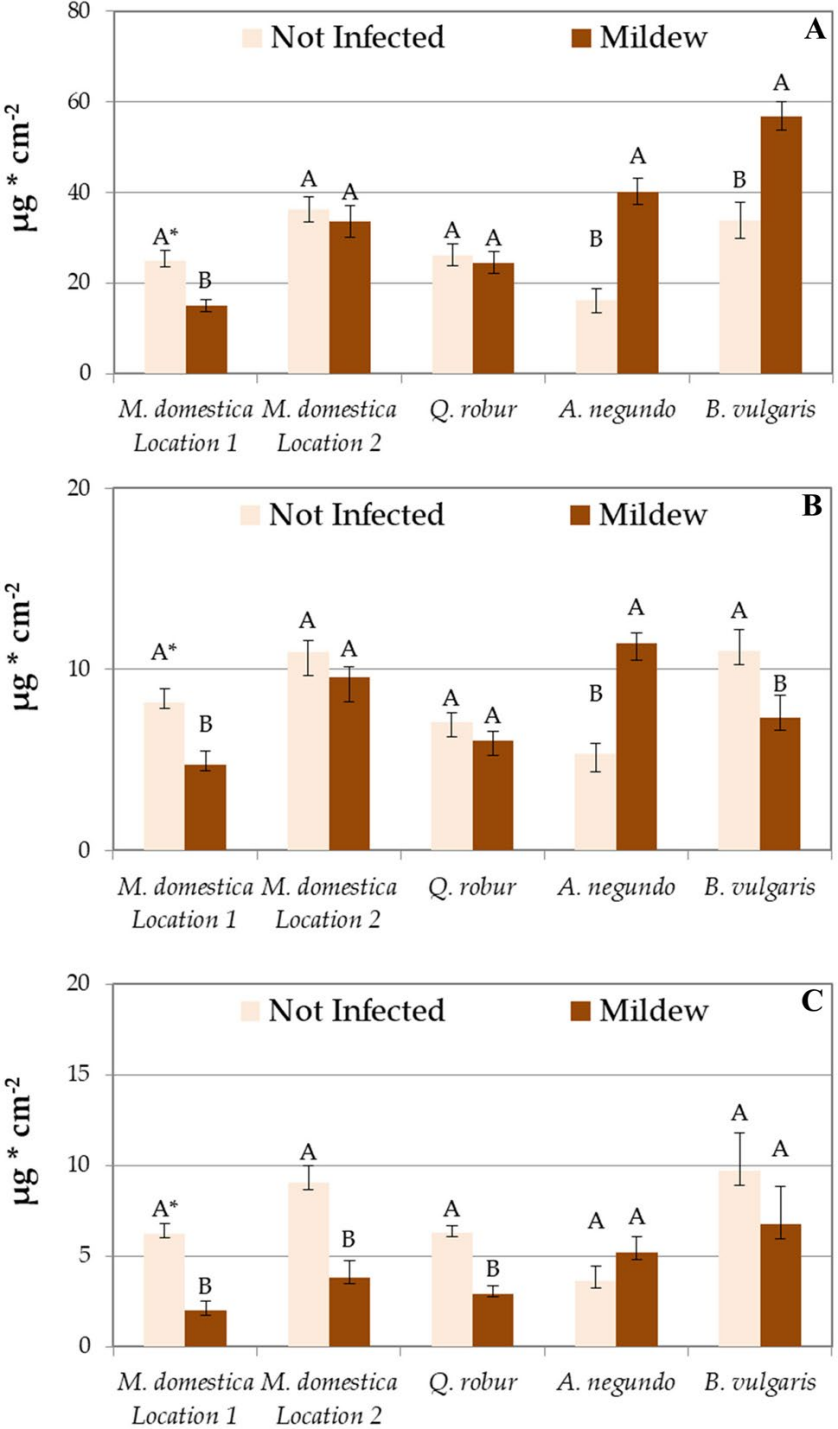
Accumulation of large (10–100 μm, **A**), coarse (2.5–10 μm, **B**), and fine (0.2–2.5 μm, **C**) PM size fractions on leaves not infected and infected with powdery mildew. Results are mean ± SE, *n* = 4 (*B. vulgaris*), 8 (*M. domestica* location 2, *Q. robur*, *A. negundo*) or 16 (*M. domestica* location 1). A single asterisk (^*^) indicates different uppercase letters indicate significant difference between leaves infected and not infected with powdery mildew at *P* < 0.05 by Tukey’s test

The leaves of *M. domestica* trees grown in location 1 not infected with powdery mildew accumulated significantly more PM of the large (10–100 μm), coarse (2.5–10 μm), and fine (0.2–2.5 μm) size fractions than fungus-infected leaves (Fig. 5). Opposite results were obtained for the *A. negundo* in which leaves infected with powdery mildew were more effective in the accumulation of all PM size fractions (significantly for fractions 10–100 μm and 2.5–10 μm). For other species and locations, recorded trends were less clear. The *M. domestica* trees from location 2 and *Q. robur* accumulated similar amounts of PM 10–100 μm and 2.5–10 μm regardless of leaf infection with powdery mildew, while the accumulation of PM 0.2–2.5 μm was higher on leaves not infected with the fungus. *B. vulgaris* accumulated more PM 10–100 μm on infected leaves, while PM 2.5–10 μm and 0.2–2.5 μm on leaves without fungus. It is noteworthy that all species except *A. negundo* accumulated; however, not always statistically significantly, more of the most dangerous PM (2.5–10 μm, 0.2–2.5 μm) on leaves not infected with powdery mildew (Fig. 5).

**Fig. 6:**
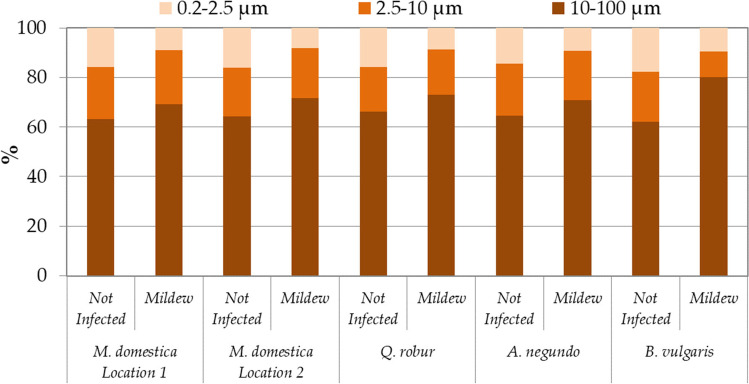
The percentage share of large (10–100 μm), coarse (2.5–10 μm), and fine (0.2–2.5 μm) PM size fractions on leaves not infected and infected with powdery mildew. Results are mean, *n* = 4 (*B. vulgaris*), 8 (*M. domestica* location 2, *Q. robur*, *A. negundo*), or 16 (*M. domestica* location 1)

Regardless of the species and location, the share of fine PM (0.2–2.5 μm) was higher on leaves not infected by powdery mildew (Fig. 6). On the contrary, the share of coarse PM (10–100 μm) was always higher on leaves infected with the fungus (Fig. 6).

**Fig. 7:**
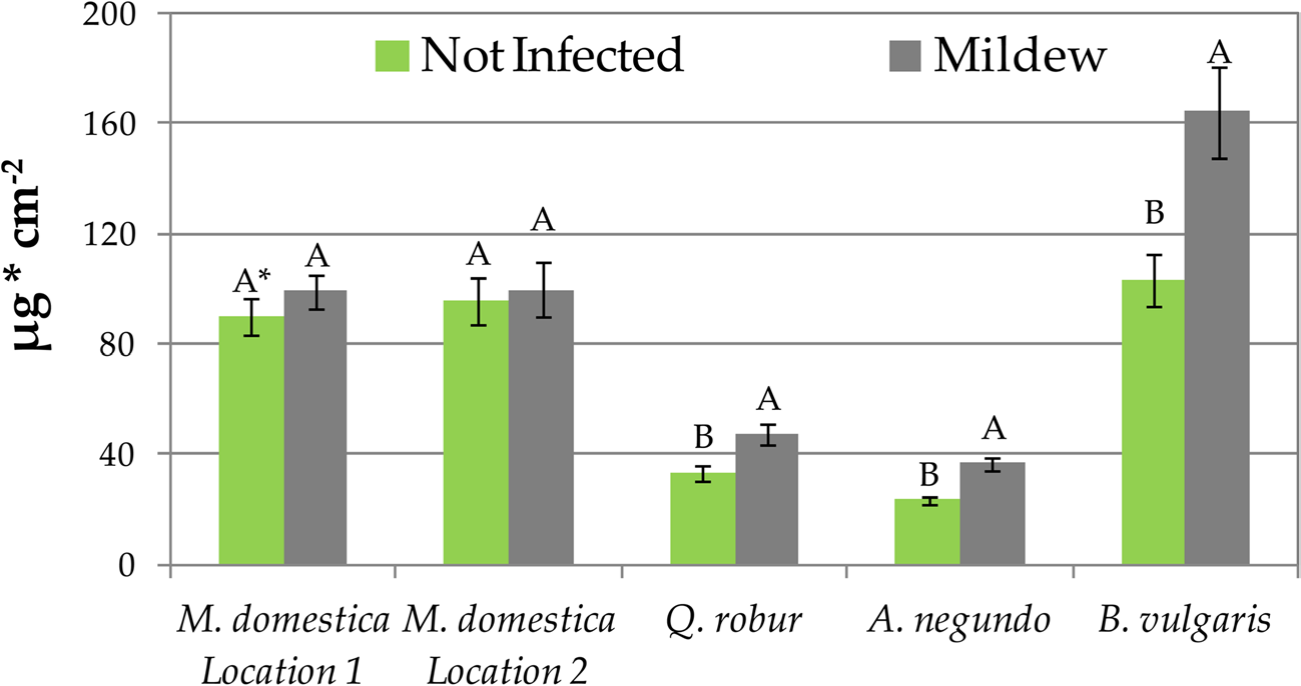
Amount of waxes on leaves not infected and infected with powdery mildew. Results are mean ± SE, *n* = 4 (*B. vulgaris*), 8 (*M. domestica* location 2, *Q. robur*, *A. negundo*), or 16 (*M. domestica* location 1). A single asterisk (^*^) indicates different uppercase letters indicate significant difference between leaves infected and not infected with powdery mildew at *P* < 0.05 by Tukey’s test

The effect of powdery mildew infection on the amount of wax on the leaf surface depended on the species examined (Fig. 7). The amount of wax on *M. domestica* leaves was the same regardless of whether the leaf was or was not infected by the fungus. In the case of *Q. robur*, *A. negundo*, and *B. vulgaris*, leaves infected by powdery mildew had significantly more wax (Fig. 7).

## Discussion

### PM accumulation on leaves infected and not infected by powdery mildew

The results obtained in this study confirm that the presence of powdery mildew, similar to nonparasitic and saprotrophic fungi, on the leaves of trees/shrubs growing in urbanized areas affects the efficiency of PM accumulation (Jouraeva et al. 2006; Mori et al. 2015). The novelty is to demonstrate that this effect is not always positive and depends on the species of the plant infected with a fungus. Until now, it was believed that the fungal mycelium always increases the complexity of the leaf surface and affects the accumulation of PM similar to the morphological structures naturally occurring on the foliage (trichomes, waxes, venation) (Mori et al. 2015; Muhammad et al. 2019). Trichomes and waxes make the leaf surface non-smooth and PM is more easily captured from the air and retained more permanently (Weerakkody et al. 2018a; Wróblewska and Jeong 2021).

The positive effect of powdery mildew mycelium on PM accumulation should be particularly pronounced on species of tree/shrub with leaves that are regular in shape and are not covered with naturally dense and long trichomes, and/or convex venation (Fröhlich-Nowoisky et al. 2009). In this study, *M. domestica* and *Q. robur* met such morphological criteria. Contrary, the presence of mycelia of the fungus on the complex, rough, and hairy leaves of *A. negundo* and *B. vulgaris* should not significantly change their biofilter potential. However, the obtained results suggest an inverse trend. The presence of powdery mildew mycelium on *M. domestica* and *Q. robur* leaves decreased the amount of PM deposited on the leaves, while increased it in the case of *A. negundo* and *B. vulgaris* foliage. The explanation may be the reduced PM retention on *M. domestica* and *Q. robur* leaves infected by powdery mildew. It is likely that PM accumulated on the mycelium covering the leaf surface is more easily removed from foliage by wind and/or rain than PM that is in direct contact with the sticky and lipophilic wax layer that is not covered with mycelium. In the case of *A. negundo* and *B. vulgaris*, this may be of less importance as these plants have a dense crown structure and complex leaves, and it is more likely that PM removed from one leaf is retained by another located lower on the plant. The influence of the fungus on the morphology of the wax is probably also of great importance. The morphological structures on the surface of the wax and its physico-chemical properties play an important role in accumulation and retention of PM on leaves (Jouraeva et al. 2006; Wróblewska and Jeong 2021). Powdery mildew mycelium may also limit the role of trichomes and rough leaf surface in PM accumulation processes. Interestingly, the morphological characteristics of the fungus had very little effect on the efficiency of PM accumulation. *M. domestica* trees were infected with powdery mildew characterized by the catenescent conidia, and as a result, the mycelium structurally resembled a PM-catching brush (like a trichome covered leaf). Nevertheless, the presence of the fungus had a negative effect on the amount of PM deposited on *M. domestica* leaves. In contrast, *B. vulgaris* covered with mildew with singly conidia (pseudoidium type) accumulated PM more efficiently when it was infected with the fungus.

It seems that the presence of fungal mycelium on the leaf surface is important for the effectiveness of plants in air biofiltration. The role of the fungus in these processes, however, is more complicated than previously thought. Further research is needed to clearly indicate in which species and under what conditions the fungus can increase the biofiltration potential of plants. Such knowledge may be of practical importance and be used during the selection of plants for planting in locations with the highest level of air pollution (e.g., near roads). Moreover, when assessing the interactions between the fungus and the accumulation of PM by plants, it should be taken into account that PM deposited on the leaf surface on the one hand affect the overall plant development and reducing the resistance of plants to drought, frost, insect, and fungal infections (Rai 2016), while on the other hand air pollution affects the biodiversity of fungi and bacteria on leaves (Stevens et al. 2021).

### Accumulation of different categories and fractions of PM by leaves infected and not infected by powdery mildew

The effectiveness of plants for PM biofiltration is assessed not only on the basis of the amount of total PM (in this work 0.2–100 μm) accumulated on foliage, but also by the amounts of fine PM (0.2–2.5 μm) and the percentage share of PM permanently retained in the wax (in-wax PM, wPM) layer (Popek et al. 2022). In this study, all plant species, regardless of whether they were infected with powdery mildew or not, accumulated the largest PM (10–100 μm) and the least fine PM. These results are consistent with previous literature reports (Jouraeva et al. 2006; Przybysz et al. 2021; Wróblewska and Jeong 2021; Popek et al. 2022). A very interesting result obtained in this work is demonstration that the percentage share of fine PM (all species) and the actual amounts of accumulated fine PM (all species except *A. negundo*) were much higher on leaves not infected by powdery mildew. The smaller the PM is, the more adverse it is to human health (Ali et al. 2019; Li and Managi 2022). Therefore, it seems reasonable that near the roads should grow plants that are healthy, not infected by powdery mildew. This will increase the chances of effective biofiltration of the most dangerous PM fraction from the air. Conversely, the percentage share of large PM was increased on leaves infected with powdery mildew, and this PM is only of little importance to human health (Ali et al. 2019).

Another important result of this research is that the percentage share of PM permanently retained in the waxes (wPM) is also usually higher on leaves not infected by powdery mildew. This phenomenon was particularly evident in *M. domestica* and *Q. robur* trees, in which the share of PM retained in wax on leaves uninfected with mildew was higher than on leaves covered with fungus. This result strengthens the previously posed hypothesis that the presence of powdery mildew mycelium on the foliage prevented the penetration of PM into the wax layer and its more permanent neutralization. PM deposited in wax is permanently inactivated, while PM deposited directly on the leaf surface can be easily removed by wind and rain, resuspended and again endanger people (Zhou et al. 2020; Wróblewska and Jeong 2021; Zhang and Ma 2021). In addition, as previously mentioned, on *M. domestica* and *Q. robur* leaves not infected by powdery mildew, the accumulation of the fine PM was significantly higher. Fine PM is not only the most dangerous PM, but also the PM which most easily penetrates deep into the waxes and inner leaf tissues. Opposite results were obtained for *A. negundo* and *B. vulgaris*; thus, two species for which the presence of powdery mildew mycelium had a positive effect on PM accumulation. In these species, the share of PM on the surface and in the waxes was the same (about 50%), regardless of the presence or absence of the fungus. Since a large part of road pollution is lipophilic (Huang et al. 2021), it can be assumed that in the absence of mycelium on the leaf surface, the share of PM retained in waxes would be higher also in case of *A. negundo* and *B. vulgaris*.

The less effective accumulation of dangerous fine PM and the reduced percentage share of PM in waxes suggests that if street trees and shrubs are to effectively contribute to air biofiltration, planting of species easily infected by powdery mildew and, most likely, other fungi should be avoided.

### Amount of waxes by leaves infected and not infected by powdery mildew

The effect of powdery mildew infection on waxes was also species-dependent. In *M. domestica* trees, no effect of fungus mycelium on amount of waxes on foliage was found, while in *Q. robur*, *A. negundo*, and *B. vulgaris*, there was significantly more wax on fungus-infected leaves; however, it is worth noting that the difference was substantial only in the case of *B. vulgaris*. The increased amount of wax on leaves infected with powdery mildew in *A. negundo* and *B. vulgaris* plants did not result in an increased share of PM deposited in wax (wPM), despite the fact that a large part of road-derived PM is lipophilic. This suggests that most probably one of the greatest consequences of the presence of fungal mycelium on the leaf surface is the reduction of the direct involvement of waxes in PM accumulation and retention processes. It seems that a detailed explanation of the effect of the mycelium covering the foliage on the wax (quantity, structure, and chemical composition) may be of key importance for assessing and understanding the effect of the fungus on the efficiency of air biofiltration by trees and shrubs.

### Limitations of the study

Our study shows that the presence of the fungus mycelium of powdery mildew on the tree/shrub foliage affects the biofiltration potential of the plants. In order to fully understand the impact of powdery mildew on PM accumulation on leaves, it is necessary to continue the research initiated in this work. In this research, plant samples were harvested one time only, in the initial period of the fungus’ appearance. This was due to the avoidance of a large amount of spores, which are the size of a large PM particle (a few to several dozen μm) and are considered to be part of PM. In order to fully understand the effect of mycelium on the accumulation of PM, it would be necessary to harvest leaves samples infected and not infected by powdery mildew in several terms, covering the entire growing season. It also seems necessary to improve the PM determination methodology in order to learn how to distinguish fungal spores from airborne PM and eliminate fungal spore them from the results. As the disease progresses (basically until the end of September), the infection spots gradually become larger and great numbers of conidia are formed (Braun 2011). Initially, in the early stages of the disease, the number of the conidia is small and, irrespective of the species, it increases as the pathogen develops. Thus, the morphological characteristics of the mycelium and foliage also change. Further research should also include more species of trees/shrubs with different leaf morphologies.

## Conclusions

The presence of powdery mildew mycelium on the foliage of trees/shrubs growing in urbanized areas affects their effectiveness in air biofiltration processes. However, the effect of the fungus on PM accumulation is species-specific. The obtained results suggest that the morphology of the leaf and the species of the fungus (e.g., different types of conidia) are of little importance for the efficiency of PM accumulation by fungus-covered leaves. The key factor is probably the mycelial coverage of the wax. As a result, the stickiness of the leaves is reduced and the accumulated PM is more easily removed from foliage by wind and rain. Leaves covered with powdery mildew accumulated less fine PM (the most dangerous to health), which suggests that trees/shrubs resistant to this pathogen should not grow along the roads.

## Data Availability

The data and materials from the current study are available from the corresponding author on reasonable request.
